# Mapping of Candidate Genes in Response to Low Nitrogen in Rice Seedlings

**DOI:** 10.1186/s12284-022-00597-x

**Published:** 2022-10-15

**Authors:** Jia Li, Wei Xin, Weiping Wang, Shijiao Zhao, Lu Xu, Xingdong Jiang, Yuxuan Duan, Hongliang Zheng, Luomiao Yang, Hualong Liu, Yan Jia, Detang Zou, Jingguo Wang

**Affiliations:** College of Agriculture, Northeast Agricultural University/Key Laboratory of Germplasm Enhancement and Physiology & Ecology of Food Crop in Cold Region, Ministry of Education, Harbin, 150030 Heilongjiang Province People’s Republic of China

**Keywords:** Rice, Genome-wide association study, RNA-seq, Low-nitrogen tolerance

## Abstract

Nitrogen is not only a macronutrient essential for crop growth and development, but also one of the most critical nutrients in farmland ecosystem. Insufficient nitrogen supply will lead to crop yield reduction, while excessive application of nitrogen fertilizer will cause agricultural and eco-environment damage. Therefore, mining low-nitrogen tolerant rice genes and improving nitrogen use efficiency are of great significance to the sustainable development of agriculture. This study was conducted by Genome-wide association study on a basis of two root morphological traits (root length and root diameter) and 788,396 SNPs of a natural population of 295 rice varieties. The transcriptome of low-nitrogen tolerant variety (Longjing 31) and low-nitrogen sensitive variety (Songjing 10) were sequenced between low and high nitrogen treatments. A total of 35 QTLs containing 493 genes were mapped. 3085 differential expressed genes were identified. Among these 493 genes, 174 genes showed different haplotype patterns. There were significant phenotype differences among different haplotypes of 58 genes with haplotype differences. These 58 genes were hypothesized as candidate genes for low nitrogen tolerance related to root morphology. Finally, six genes (*Os07g0471300*, *Os11g0230400*, *Os11g0229300*, *Os11g0229400*, *Os11g0618300* and *Os11g0229333*) which expressed differentially in Longjing 31 were defined as more valuable candidate genes for low-nitrogen tolerance. The results revealed the response characteristics of rice to low-nitrogen, and provided insights into regulatory mechanisms of rice to nitrogen deficiency.

**Supplementary Information:**

The online version contains supplementary material available at 10.1186/s12284-022-00597-x.

## Background

Rice (*Oryza sativa* L.) is one of the three major food crops in the world. More than 50% of the world's population rely on rice as their main food source (Zibaee 2013). At present, the high yield of crops in many countries and regions mainly depends on the application of chemical fertilizers, especially nitrogen fertilizer. Nitrogen is an essential nutrient element affecting plant growth, development and final yield formation (Lawlor 2002). Although the application of nitrogen fertilizer has greatly increased the yield of rice, it has also led to problems such as the reduction of nitrogen use efficiency (Purwani et al. 2021), the aggravation of water eutrophication (Yin et al. 2007) and soil hardening (Meng et al. 2005), which have brought enormous pressure to the ecological environment. How to ensure high yield on the premise of reducing the amount of nitrogen fertilizer has become one of the main challenges faced by rice breeding researchers.

Root system has the functions of fixing plants, absorbing nutrients and water, and synthesizing some physiologically active substances, and is necessary for plant growth and development (Karthika et al. 2018). In recent years, more and more researches have focused on studying the effect of nitrogen on root system. The nitrogen supply level significantly affects the root architecture and physiological characteristics of rice (Kant et al. 2011). Nitrogen absorption and nitrogen utilization are two main factors affecting nitrogen use efficiency (Latshaw et al. 2016). Root length and root diameter were the most significant positive and negative correlation factors affecting nitrogen absorption efficiency (Ren et al. 2013). Root activity was closely related to nitrogen absorption, and a reasonable nitrogen ratio was conducive to the improvement of runoff intensity (Xiong et al. 2016). An effective solution to improve yield yet free of pollution is the reduction of nitrogen fertilizer and the improvement of nitrogen use efficiency.

In order to adapt to the nitrogen-deficient environment, plants activate a "foraging response" of their own roots, and change root structure to further affect the perception and absorption of nitrogen in the rhizosphere micro-environment (Lawlor 2005). Low-nitrogen tolerant variety can not only efficiently absorb nitrogen in soil, but also effectively utilize the absorbed nitrogen to form yield. At present, researchers have found that increased yield was largely given to the improvement of crop tolerance to nitrogen stress, and the heritability of crop nitrogen use efficiency under low-nitrogen condition was significantly greater than that under high-nitrogen condition (Tollenaar and Wu 1999; Zou et al. 2008; Liu et al. 2013). In addition, scientists have identified and cloned some genes that are expected to significantly improve rice yield and nitrogen use efficiency, such as *DEP1* (Huang et al. 2009), *GRF4* (Duan et al. 2015), *NRT1.1B* (Fan et al. 2016), *NR2* (Gao et al. 2019) and *NAC42-NPF6.1* (Tang et al. 2019). Therefore, it is effective to alleviate crop nitrogen deficiency by fully exploiting the abundant plant germplasm resources, screening low-nitrogen tolerant crop genotypes, and cultivating low-nitrogen tolerant varieties through genetic manipulation.

With the advent of next-generation sequencing, genome-wide association study (GWAS) that aims to identify direct associations between genotype and phenotype in a natural population has rapidly become a powerful gene/QTL mapping tool (Zhang et al. 2012; Korte and Farlow 2013; Kim and Reinke 2019; Han et al. 2018; Zhou et al. 2019). GWAS has been successfully used to identify QTLs associated with disease resistance (Wang et al. 2015), abiotic stress (Challa and Neelapu, 2018), yield (Liu et al. 2018) and other traits in rice. Transcriptome has also been applied to various aspects of plant biology, such as the clarifying signal transduction mechanism of drought stress response in Arabidopsis (Harb et al. 2010) and excavating genes involved in adult resistance of wheat (El et al. 2021). Genes related to nitrogen metabolism in crop roots such as wheat (Guo et al. 2014), common beans (Nanjareddy et al. 2017), pears (Chen et al. 2018) and potatoes (Zhang et al. 2020) under nitrogen treatment had been identified through transcriptome analysis. The genetic mechanisms of many complex traits have been analyzed by integrating GWAS and RNA-seq. For example, 39 salt-responding differential expressed genes (DEGs) related to signal regulation of salt tolerance, including *CYPs*, *LRR-KISS* and *CML* were verified in barley (Tu et al. 2021); sixteen candidate genes of seed fatty acid metabolism and protein synthesis of cultivated peanut were screened (Zhang et al. 2021b); five DEGs were identified as promising candidate genes between long and short maize roots at seedling stage (Wang et al. 2019).

In this study, GWAS and RNA-seq were combined to identify candidate genes of low-nitrogen tolerance in major QTLs interval. Root length (RL) and root diameter (RD) of 295 rice varieties were measured under low and high nitrogen treatments at seedling stage. GWAS was performed based on phenotype (RL and RD) and 788,396 single-nucleotide polymorphisms (SNPs) developed by re-sequencing 295 rice varieties. Two rice varieties showing significant phenotypic differences of root under low-nitrogen treatment were chosen to conduct RNA-seq. Furthermore, six low-nitrogen tolerance-related candidate genes were obtained by haplotype and expression analysis. Results of this study have provided theoretical basis for rapid exploration of low-nitrogen response genes and the improvement of low-nitrogen tolerance in rice.

## Results

### Phenotypic Data Analysis

**Fig. 1:**
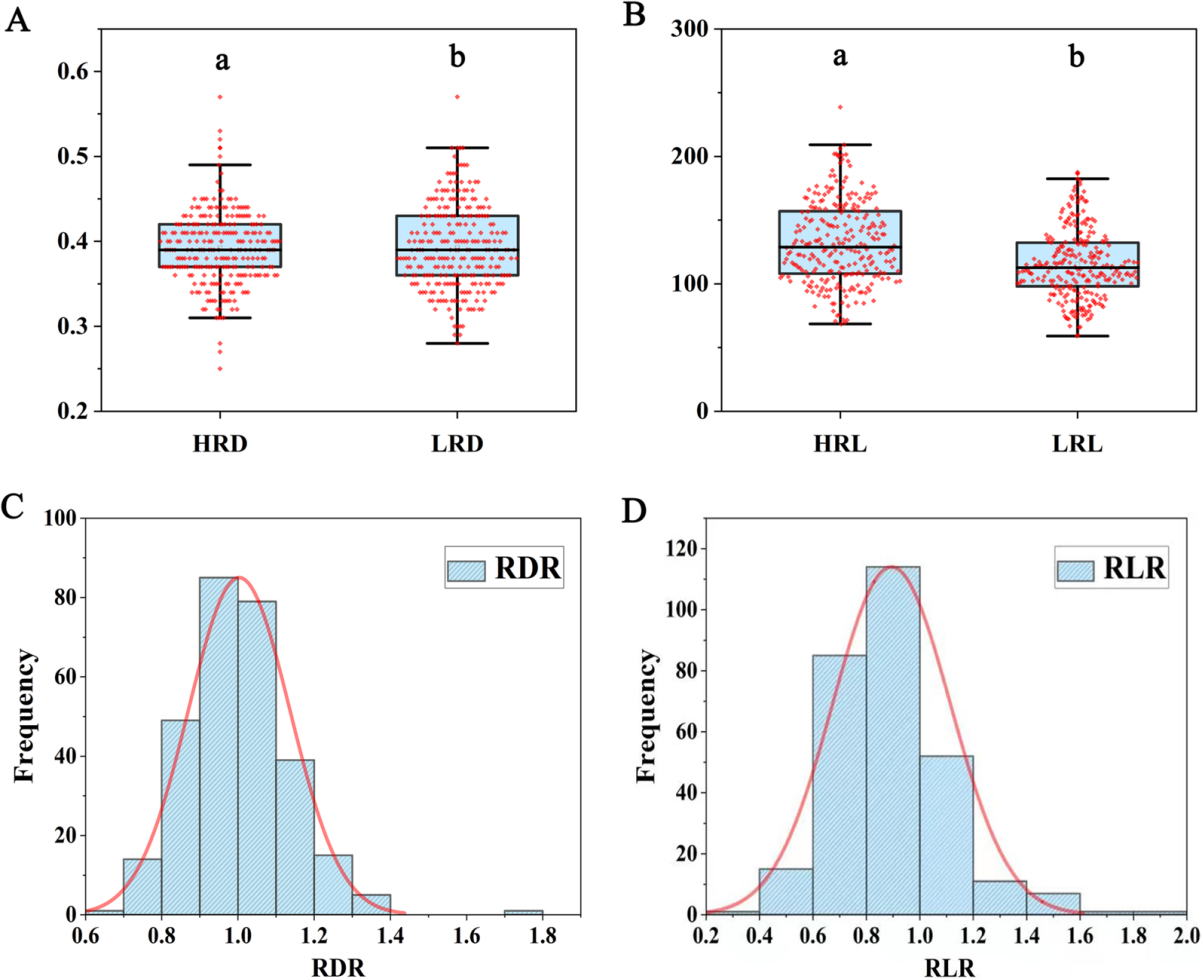
Phenotype distribution of root morphology traits of 295 rice varieties under low and high nitrogen treatments. **A** HRD: root diameter under high-nitrogen treatment. LRD: root diameter under low-nitrogen treatment. **B** HRL: root length under high-nitrogen treatment. LRL: root length under low-nitrogen treatment. **C** RDR: relative value of root diameter under low and high nitrogen treatments. **D** RLR: relative value of root length under low and high nitrogen treatments

In order to evaluate phenotypic variation of nitrogen uptake in 295 rice accessions, the root length (RL) and root diameter (RD) at seedling stage were measured under low and high nitrogen treatments. The mean value, interval, standard deviation and coefficient of variation of the root morphology traits (RL and RD) are shown in Additional file 2: Table S1. Under low and high nitrogen treatments, the coefficients of variation ranged from 12.25 to 23.53 and 11.07 to 24.15, respectively. Among the trait relative values, the relative value of root diameter (RDR) under low and high nitrogen treatments showed the smallest coefficient of variation. RL of high-nitrogen treatment was higher than that of low-nitrogen treatment. Abundant genetic variations were displayed for each trait in association panel and all phenotypic values were approximately normally distributed (Fig. 1, Additional file 1: Fig. S1). Significant correlations were observed between RL and RD whether under low or high nitrogen treatment, but the correlation between RDR and the relative value of root length (RLR) under low and high nitrogen treatments was not significant (Additional file 3: Table S2).

### QTLs Identification

**Fig. 2:**
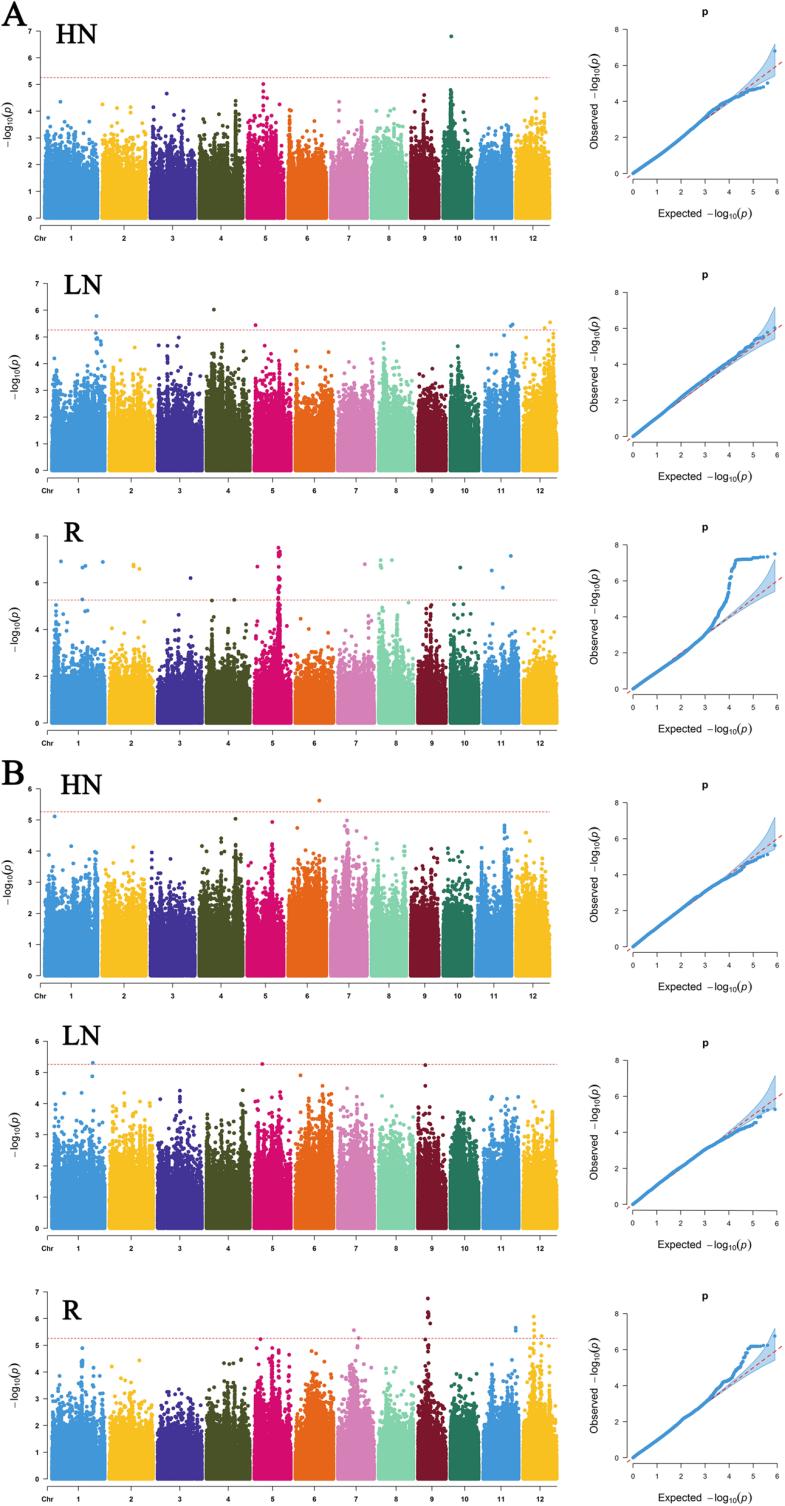
Manhattan plots and quantile–quantile (Q–Q) plots of root morphology traits. **A** GWAS assays of root diameter under high and low nitrogen treatments. **B** GWAS assays of root length under high and low nitrogen treatments. LN: low-nitrogen. HN: high-nitrogen. R: relative value of traits under low and high nitrogen treatments

The Manhattan and Q–Q plots of the GWAS results are shown in Fig. 2. GWAS identified 35 leading SNPs in the whole panel (Additional file 4: Table S3). Five significant leading SNPs associated with LRD were identified on chromosomes 1, 4, 5, 11, 12 and named *qLRD1*, *qLRD4*, *qLRD5*, *qLRD11* and *qLRD12*, respectively. One significant leading SNP associated with HRD was detected on chromosomes 10 and named *qHRD10*. Two significant leading SNPs associated with LRL were mapped on chromosomes 1, 5 and named *qLRL1* and *qLRL5*, respectively. One significant leading SNP associated with HRL was identified on chromosomes 6 and named *qHRL6*. Nineteen significant leading SNPs associated with RDR were detected on chromosomes 1, 2, 3, 4, 5, 7, 8, 10, 11 and named *qRDR1-1*, *qRDR1-2*, *qRDR1-3*, *qRDR1-4*, *qRDR2-1*, *qRDR2-2*, *qRDR3*, *qRDR4*, *qRDR5-1*, *qRDR5-2*, *qRDR7*, *qRDR8-1*, *qRDR8-2*, *qRDR8-3*, *qRDR8-4*, *qRDR10*, *qRDR11-1*, *qRDR11-2* and *qRDR11-3*, respectively. Seven significant leading SNPs associated with RLR were mapped on chromosomes 7, 9, 11, 12 and named *qRLR7-1*, *qRLR7-2*, *qRLR9-1*, *qRLR9-2*, *qRLR11*, *qRLR12-1* and *qRLR12-2*, respectively (Fig. 2 and Additional file 4: Table S3). There were 493 genes in the 35 QTL intervals (Additional file 5: Table S4).These leading SNPs are essential for rice in mediating root growth and development in response to nitrogen at seedling stage.

### Candidate Gene Mining

Among these 493 genes, 174 genes showed different haplotype patterns, with 96 genes having 2 haplotypes, 54 genes having 3 haplotypes, 18 genes having 4 haplotypes, 4 genes having 5 haplotypes, and 2 genes having 6 haplotypes, respectively. According to the phenotypic data of 295 accessions, there were significant phenotype differences among different haplotypes of 58 genes (Additional file 5: Table S4). Therefore, we hypothesized that these 58 genes were candidate genes for low nitrogen tolerance related to root morphology.

**Fig. 3:**
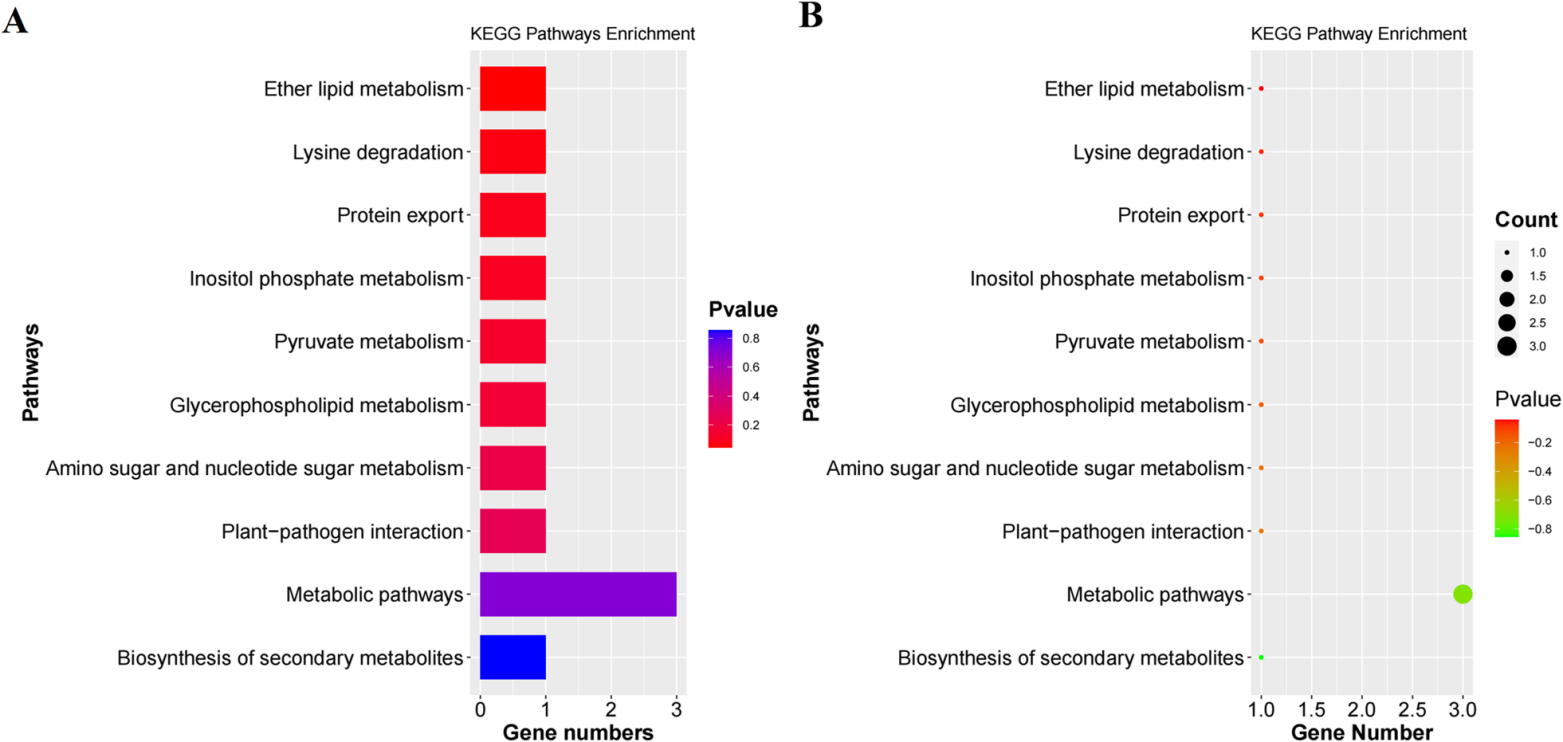
KEGG functional classification and biological pathway enrichment of 58 genes

**Fig. 4:**
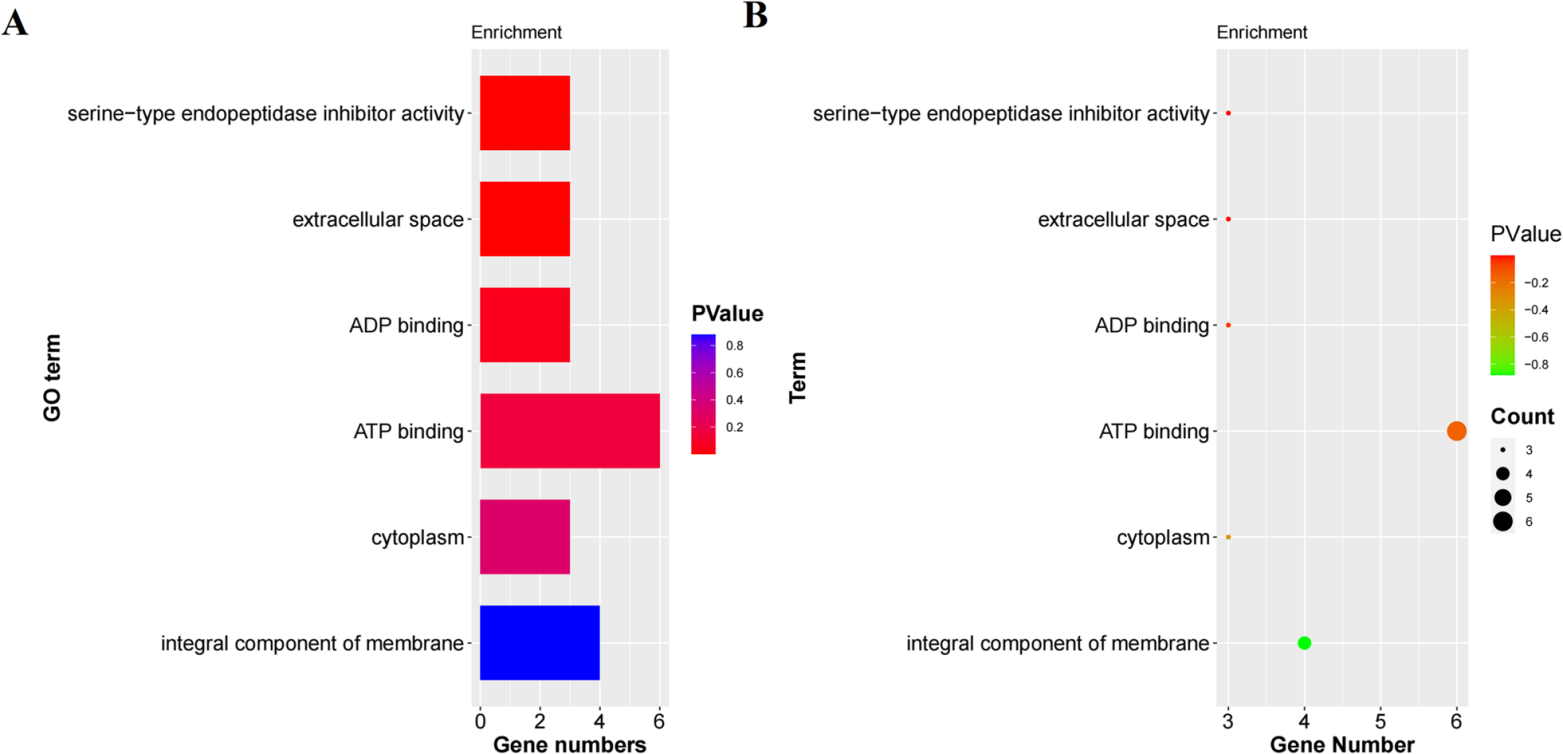
GO classification analysis of 58 genes

KEGG enrichment analysis and GO classification analysis were conducted for these 58 genes (Figs. 3, 4). These 58 genes were mainly distributed in the metabolic category. The number of gene in metabolic pathways is the most (3 genes). A total of 6 functional groups were annotated among these 58 genes, including 3 cellular component categories and 3 molecular function categories. There were 6 genes in the molecular function of ATP binding.

### RNA-Seq Statistics

**Fig. 5:**
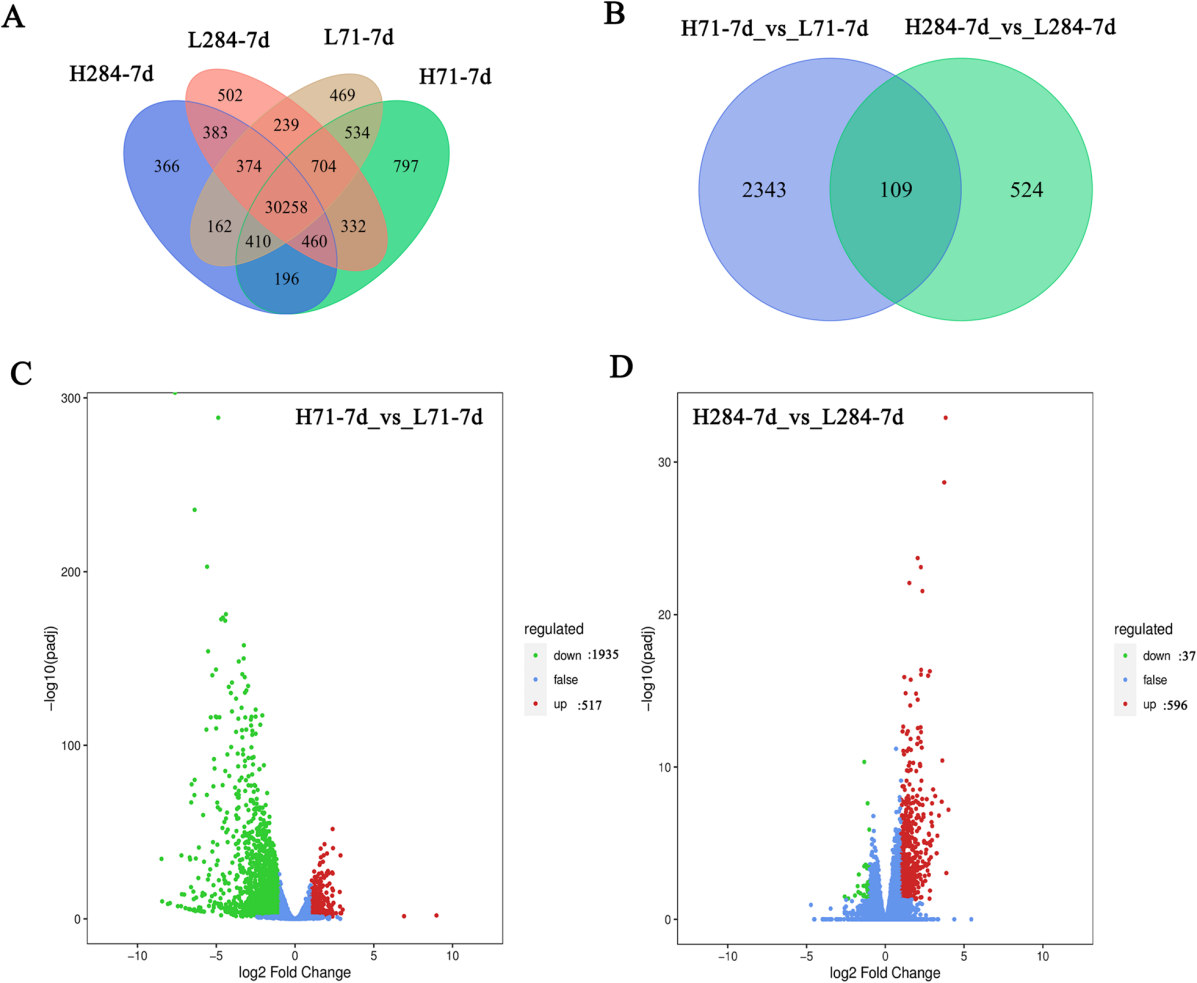
The differential expressed genes (DEGs) in roots. **A** Venn diagram of DEGs among different samples. **B** Venn diagram of DEGs between two comparative groups. **C** and **D** The volcano plots of two comparative groups. X-axis and Y-axis present the value of log_2_^(ratio)^ and −log_10_^(FDR)^ of two comparative groups, respectively. Red (Up regulated) and green (down regulated) dots indicated that the genes have significant expression difference, while the blue dots represent genes with no significant differences. H71-7d: The samples of Longjing 31 treated under high-nitrogen treatment for 7 days. L71-7d: The samples of Longjing 31 treated under low-nitrogen treatment for 7 days. H284-7d: The samples of Songjing 10 treated under high-nitrogen treatment for 7 days. L284-7d: The samples of Songjing 10 treated under low-nitrogen treatment for 7 days

The transcriptome analysis was conducted to clarify the expression differences of these 58 genes (Additional file 6: Table S5). Two comparative groups (H71 vs. L71 and H284 vs. L284) were set up for the two rice cultivar under different treatments. The letter H, letter L, number 71 and number 284 were designated to represent high-nitrogen, low-nitrogen, Longjing 31 (low-nitrogen tolerant variety) and Songjing 10 (low-nitrogen sensitive variety), respectively. As shown in Fig. 5A, gene expression levels differed greatly among different treatments and among different varieties. There were 2452 and 633 significant differential expressed genes (DEGs) in the two comparative groups, indicating significant differences of the gene expression level within the varieties and the treatments (Fig. 5B). Among the 2452 DEGs between the treatment (low-nitrogen) and control (high-nitrogen) of Longjing 31, 517 genes were up-regulated and 1935 genes were down-regulated. The number of up-regulated and down-regulated DEGs was 596 and 37 respectively, among the 633 DEGs between the treatment and control of Songjing 10 (Fig. 5C and D). Obviously, the DEGs quantity of Longjing 31 was more than that of Songjing 10. This result indicated that the effect of nitrogen stress on the low-nitrogen tolerant variety was greater than that on the low-nitrogen sensitive variety. Among these 3085 DEGs, 14 genes were associated with nitrogen utilization (Additional file 7: Table S6) according to the Rice Genome Annotation Project (http://rice.plantbiology.msu.edu/). These 14 DEGs included 7 different types, which were peptide transporter, nitrate reductase, nitrate transporter, POT family protein, proton-dependent oligopeptide transport, ammonium transporter, and NADH-glutamate synthase.

**Fig. 6:**
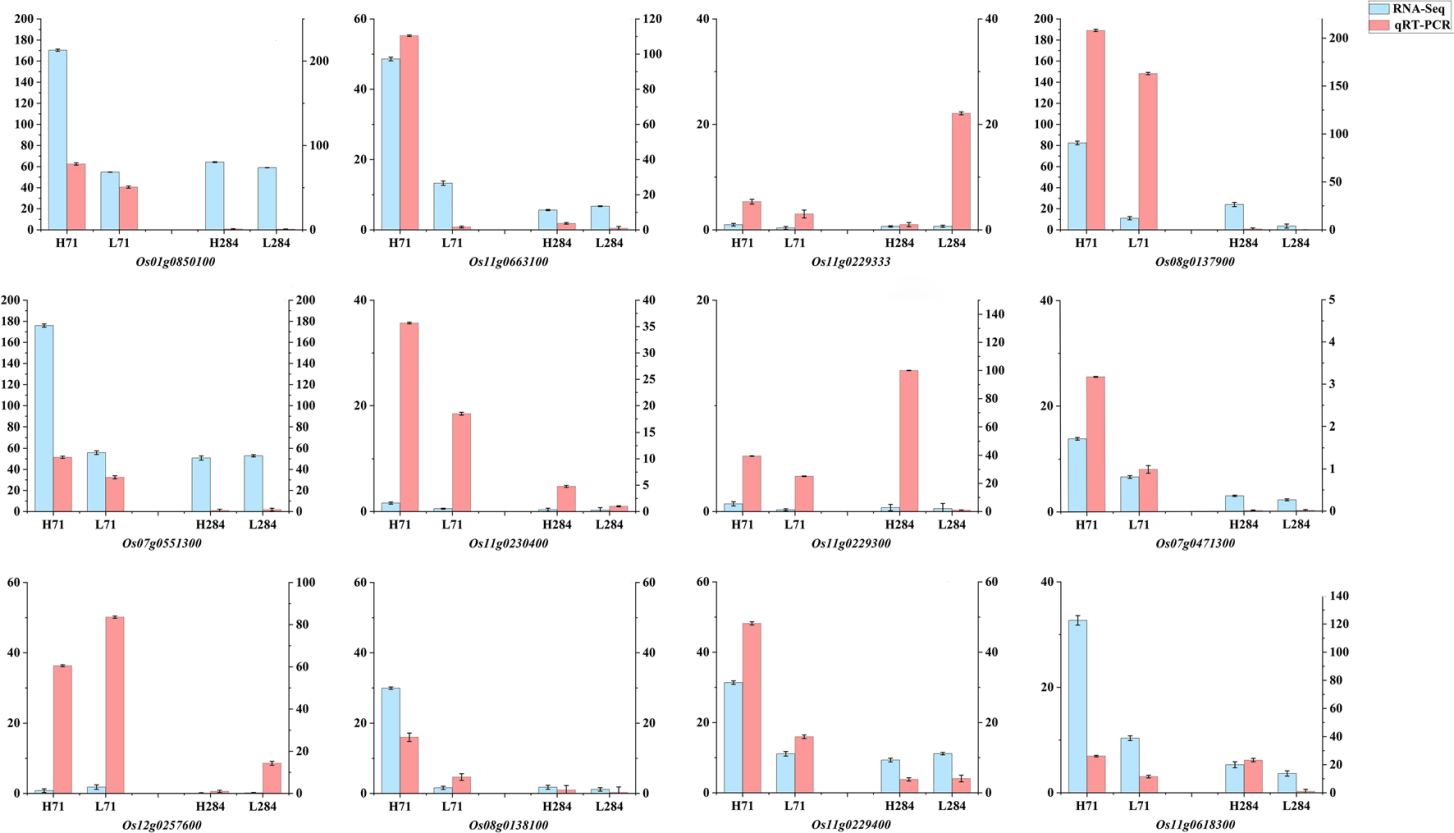
qRT-PCR analysis of gene expression levels. H71: Longjing 31 under high-nitrogen treatment. L71: Longjing 31 under low-nitrogen treatment. H284: Songjing 10 under high-nitrogen treatment. L284: Songjing 10 under low-nitrogen treatment

To confirm the accuracy and reproducibility of Illumina RNA-Seq results, 12 genes were randomly selected to verify their expression levels between Longjing 31 and Songjing 10 under low and high nitrogen treatments by qRT-PCR. Relative expression trends of these 12 genes were consistent between the qRT-PCR results and RNA-seq data, but the absolute expression levels showed some differences (Fig. 6).

### Expression Analysis of Candidate Genes

**Fig. 7:**
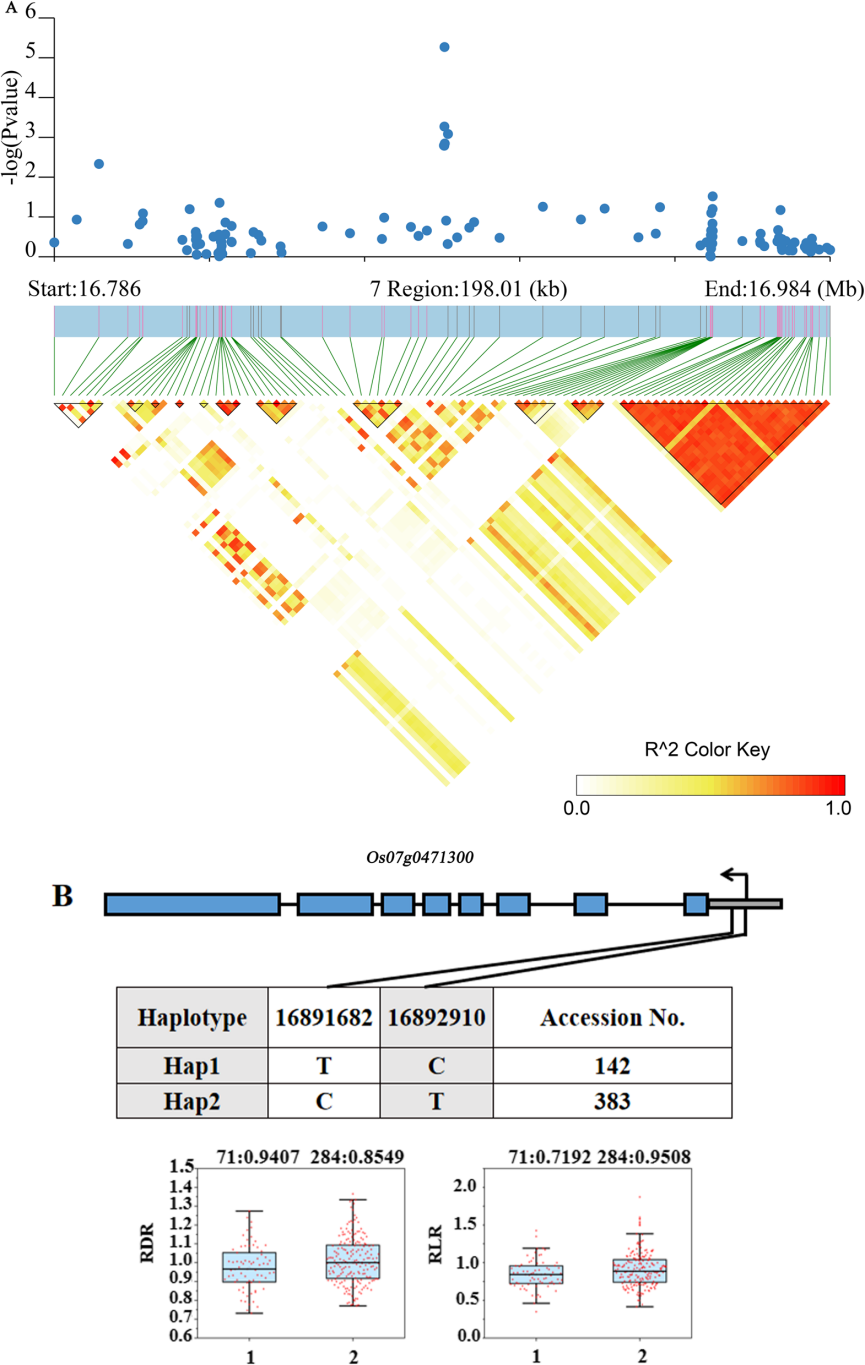
Associated region and haplotype analysis of *Os07g0471300*. A, Regional Manhattan plots and LD heatmap of *Os07g0471300*. B, Gene structure and haplotype analysis of *Os07g0471300*. RDR: relative value of root diameter under low and high nitrogen treatments. RLR: relative value of root length under low and high nitrogen treatments

**Fig. 8:**
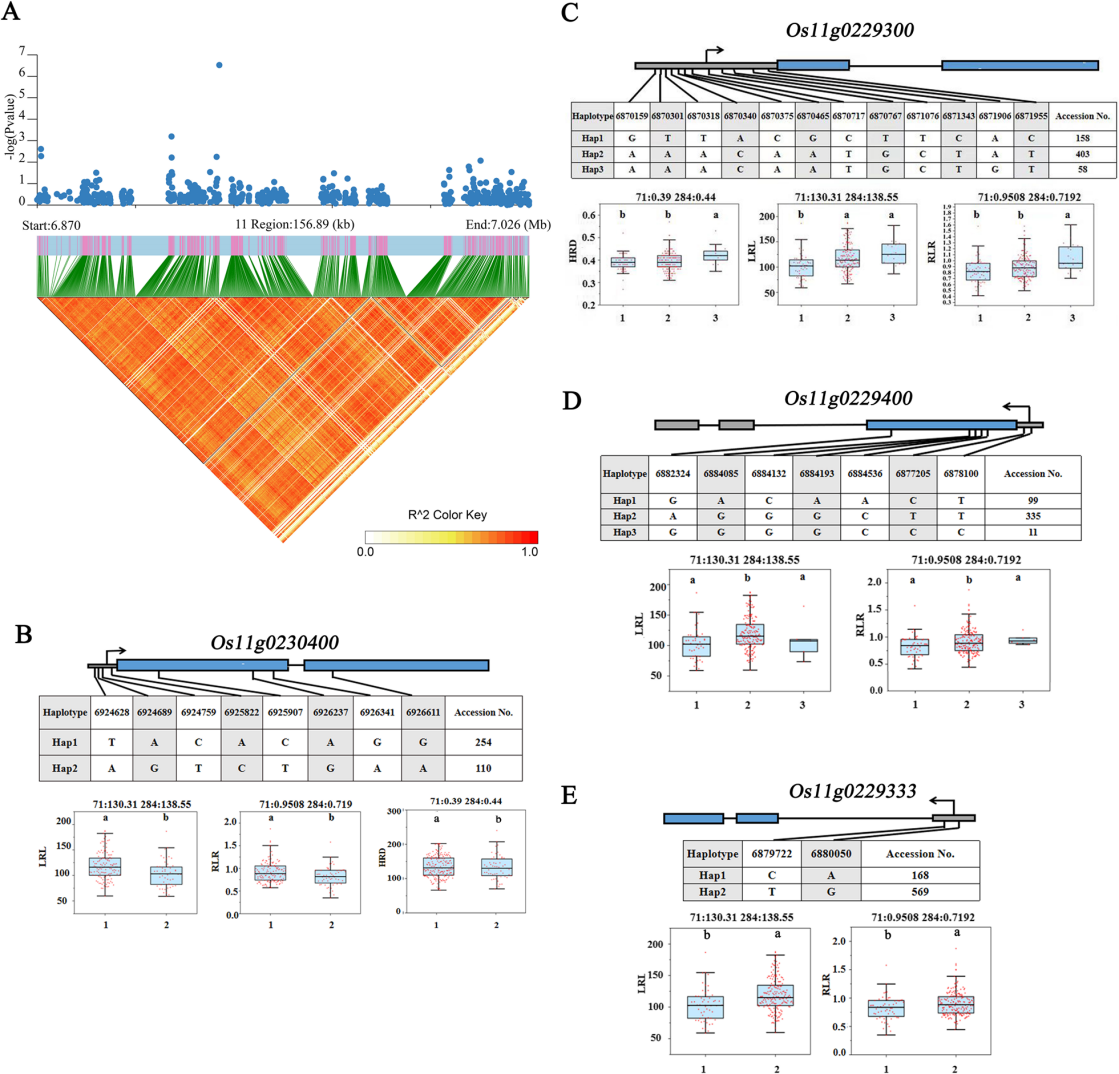
Associated region and haplotype analysis of *Os11g0230400*, *Os11g0229300*, *Os11g0229400* and *Os11g0229333*. **A** Regional Manhattan plots and LD heatmap of *Os11g0230400*, *Os11g0229300*, *Os11g0229400* and Os11g0229333. **B** Gene structure and haplotype analysis of *Os11g0230400*. **C** Gene structure and haplotype analysis of *Os11g0229300*. **D** Gene structure and haplotype analysis of *Os11g0229400*. **E** Gene structure and haplotype analysis of *Os11g0229333*. LRL: root length under low-nitrogen treatment. RLR: relative value of root length under low and high nitrogen treatments. HRD: root diameter under high-nitrogen treatment

**Fig. 9:**
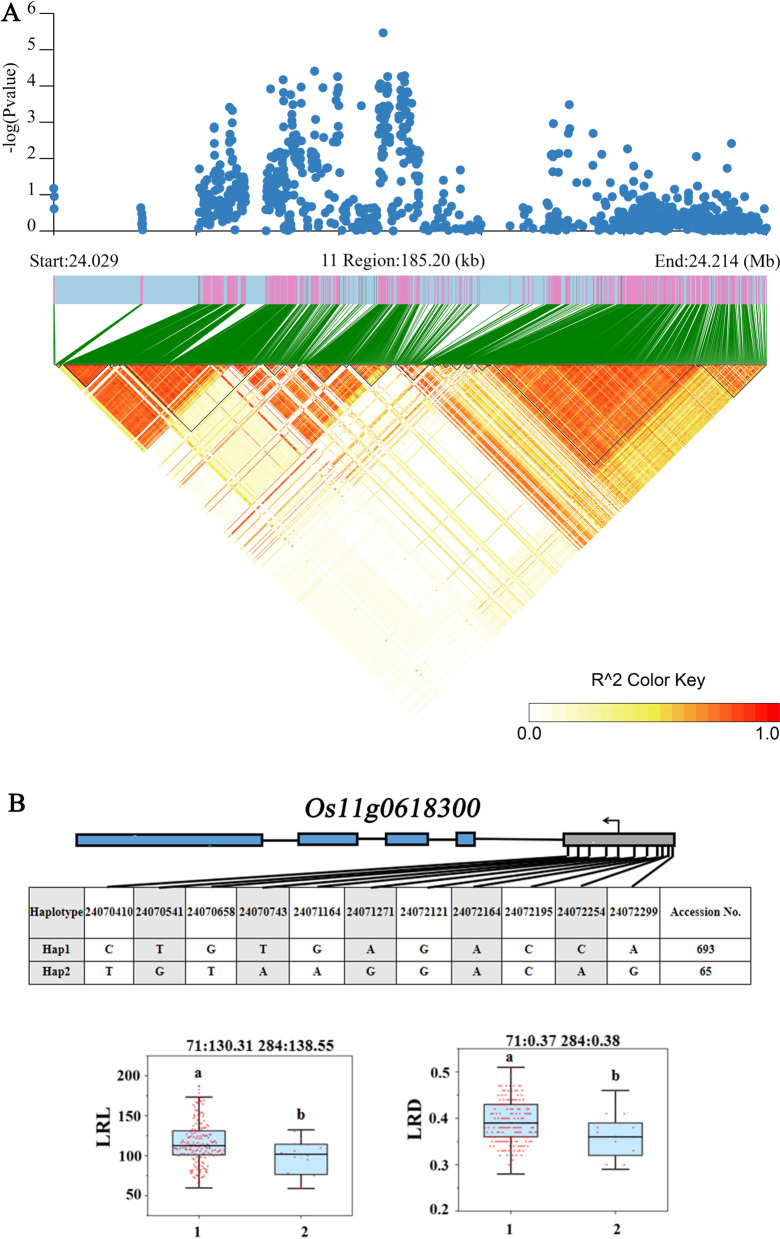
Associated region and haplotype analysis of *Os11g0618300*. **A** Regional Manhattan plots and LD heatmap of *Os11g0618300*. **B** Gene structure and haplotype analysis of *Os11g0618300*. LRD: root diameter under low-nitrogen treatment. LRL: root length under low-nitrogen treatment

According to the results of transcriptome sequencing, six genes were down-regulated and four genes were up-regulated between the treatment (low-nitrogen) and control (high-nitrogen) of Longjing 31 and Songjing 10, respectively (Additional file 8: Table S7). Therefore, these 6 genes which expressed differentially in Longjing 31, *Os07g0471300*, *Os11g0230400*, *Os11g0229300*, *Os11g0229400*, *Os11g0618300* and *Os11g0229333,* were defined as more valuable candidate genes among the 58 genes with haplotype differences for this study (Table 1). The linkage disequilibrium (LD) of these six candidate genes were showed in Figs. 7A, 8A and 9A. Two SNPs located in the promoter region of *Os07g0471300* formed two haplotypes, while no nonsynonymous SNP or InDel was found in CDS (Fig. 7B). In the two haplotypes of *Os11g0230400*, a total of 4 SNPs were detected in the promoter region, and 4 nonsynonymous SNPs or InDels were found in CDS (Fig. 8B). Three haplotypes of *Os11g0229300* included 12 SNPs in the promoter region (Fig. 8C). The haplotype analysis of *Os11g0229400* indicated that 2 SNPs were identified in the promoter region and 5 nonsynonymous SNPs or InDels were detected in CDS (Fig. 8D). Two haplotypes of each gene were defined by 2 and 11 SNPs located in the promoter region of *Os11g0229333* and *Os11g0618300*, respectively (Figs. 8E and 9B).

**Table 1:** Candidate genes expressed differentially under low nitrogen conditions

Candidate genes	Regulated	QTLs	Annotation
*Os07g0471300*	Down	*qRLR7-2*	*AGO* protein
*Os11g0230400*	Down	*qRDR11-1*	Serpin domain containing protein, putative, expressed
*Os11g0229300*	Down	*qRDR11-1*	Disease resistance protein *RPM1*, putative, expressed
*Os11g0229400*	Down	*qRDR11-1*	Disease resistance protein *RPM1*, putative, expressed
*Os11g0618300*	Down	*qLRD11*	Protein kinase family protein, putative, expressed
*Os11g0229333*	Down	*qRDR11-1*	Hypothetical gene

## Discussion

Nitrogen is an important factor regulating growth and development of rice root system. To promote plants absorption of nitrogen can help reduce the application of fertilizers, which is a very important factor in cutting agricultural costs, controlling pollution and ensuring food safety. Plants have evolved with various response strategies to low-nitrogen stress so as to complete their life cycles and reproduce offspring in nitrogen deficient environment. The study also found that crop adaptation to low-nitrogen was positively correlated to yield (Kosgey et al. 2013). Therefore, the genetic improvement of crops low-nitrogen tolerance is not only feasible in theory, but also practical in reality. Roots play an essential role in the absorption of water and nutrients. Understanding the genetic basis of rice root development under low-nitrogen condition is conducive to breeding new varieties with low-nitrogen tolerance and enhancing resource utilization efficiency. Different ways of nitrogen treatment will directly affect the development of rice root. Previous studies showed that low-nitrogen condition had improved root morphology traits of rice and maize, such as the total root length, main root length, lateral root length and the ratio of root dry weight to whole plant dry weight (Chun et al. 2005; Wang et al. 2005; Potarzycki 2011). In this study, the effects of low-nitrogen on root morphology traits were studied by measuring root length and root diameter.

Most of the previous mapping studies were based on classical QTL mapping (Sudarshan et al. 2017; Zhang et al. 2021a; Singh et al. 2021; Weng et al. 2008; Wan et al. 2006; Maeda et al. 2004), which utilizes the limited genetic variations between two specific parents. However, GWAS takes advantage of extensive variations in natural population (Wang et al. 2020a). In the past decade, GWAS has successfully mapped plenty of potential loci in rice and identified several critical genes, which have been further conformed in subsequent functional experiments (Chen et al. 2014; Si et al. 2016; Yano et al. 2016). This study was conducted by Genome-wide association study on a basis of two root morphological traits (root length and root diameter) and 788,396 SNPs of a natural population of 295 rice varieties. There were 493 genes in the 35 QTL intervals identified in this study. Among these 493 genes, 58 genes with haplotype differences had significant phenotypic differences among all samples (Additional file 5: Table S4). Therefore, we hypothesized that these 58 genes were candidate genes for low nitrogen tolerance related to root morphology.

The genome-wide investigation of gene expression using RNA-seq is an effective approach to analyze gene regulatory networks of the complex agronomic trait. Transcriptome analysis can be used to characterize the response of plant to environmental stresses (Wang et al. 2009). Under low-nitrogen condition, many genes show differential expressions, which are crucial in rice adaptation to low-nitrogen stress (Subudhi et al. 2020). Through transcriptome sequencing, 3085 differential expressed genes (DEGs) were identified between the treatment (low-nitrogen) and control (high-nitrogen) of Longjing 31 and Songjing 10. Among these 3085 DEGs, 14 genes were associated with nitrogen utilization (Additional file 7: Table S6) according to the Rice Genome Annotation Project (http://rice.plantbiology.msu.edu/). Among the 14 genes, 6 genes have been studied completely by predecessors. *OsNRT2.1* (*Os02g0112100*) encoded a high-affinity nitrate transporter, highly expressed throughout roots and mainly functioned in nitrate uptake under low nitrate concentrations (Feng et al. 2011). *OsPTR6* (*Os04g0597800*) encoded a small peptide transporter and expressed in both roots and stems of over-expressed plants. The up-regulate expression of *OsPTR6* could be activated by the increase of NO_3_^−^ concentration in roots (Fan et al. 2014). *OsNPF4.5* (*Os01g0748950*) encoded a nitrate transporter. Increasing the expression abundance of *NPF4.5* can significantly improve the nitrogen uptake efficiency and promote rice growth (Wang et al. 2020b). *OsNRT2.2* (*Os02g0112600*) encoded a high-affinity nitrate transporter. The expression of *OsNRT2.2* was promoted by nitrate and inhibited by NH_4_^+^ at a high temperature of 37 °C (Feng et al. 2011). It is reported that *OsAMT1;3* (*Os02g0620500*, encoding an ammonium transporter) regulated the uptake of ammonium nitrogen cooperatively under low-nitrogen condition (Konishi and Ma, 2021). *OsNADH-GOGAT2* (*Os05g0555600*) is a NADH-glutamate synthase gene. According to previous reports, the total nitrogen content of senescent leaves of *OsNADH-GOGAT2* mutant is only about half of that of wild type plant (Tamura et al. 2011). Among the 14 genes, 8 genes have not been studied thoroughly by predecessors. They may have the function of affecting rice nitrogen use efficiency, which is worthy of further researches.

Low-nitrogen tolerance is a complex trait controlled by multiple genes. In recent years, many low-nitrogen tolerance genes have been identified, such as *TOND1*, *OsNAC42*, *OsNPF6.1* and *OsTCP19* (Zhang et al. 2015; Tang et al. 2019; Liu et al. 2021). With the development of high-throughput sequencing technology, the research method combining GWAS and RNA-seq has provided possibilities to quickly analyze the genetic basis of complex traits, such as lncRNAs response to low-nitrogen, seed germination ability and seedlings root length under drought stress (Guo et al. 2020; Ma et al. 2022). Our previous research also identified many saline-alkaline tolerant candidate genes (*OsIRO3*, *OsSAP16*) by integrating GWAS/BSA and transcriptome data analysis (Li et al. 2019; Lei et al. 2020). In this study, the transcriptome of low-nitrogen tolerant variety and low-nitrogen sensitive variety were sequenced. By combining the amount of gene expression, six genes which expressed differentially in low-nitrogen tolerant varietie were defined as more valuable candidate genes among the 58 genes with haplotype differences (Table 1). Haplotype analysis revealed that these genes showed different haplotype patterns in association panel. There were significant phenotype differences among different haplotypes of these genes. As far as we know, there is no report on the breeding value of these six candidate genes. The results showed six favorable alleles including Hap2 of *Os07g0471300*, Hap1 of *Os11g0230400*, Hap3 of *Os11g0229300*, Hap2 of *Os11g0229400*, Hap1 of *Os11g0618300* and Hap2 of *Os11g0229333* (Figs. 7, 8, 9). The combination of GWAS and transcriptome was proved to be an efficient method to mine favorable alleles in improved varieties.

## Conclusion

In this study, GWAS was conducted on a basis of two root morphological traits (root length and root diameter) and 788,396 SNPs of a natural population of 295 rice varieties. The transcriptome of low-nitrogen tolerant variety (Longjing 31) and low-nitrogen sensitive variety (Songjing 10) were sequenced between low and high nitrogen treatments. There were 493 genes in the 35 QTL intervals mapped in this study. 3085 differential expressed genes were identified. Haplotype analysis revealed that 174 out of the 493 genes showed different haplotype patterns in the association panel. There were significant phenotype differences among different haplotypes of 58 genes with haplotype differences. These 58 genes were hypothesized as candidate genes for low nitrogen tolerance related to root morphology. The expression levels of these 58 genes were analyzed and six genes which expressed differentially in low-nitrogen tolerant variety were defined as more valuable candidate genes among the 58 genes. However, further study was needed to investigate the regulation mechanism of these candidate genes mediating low-nitrogen tolerance in rice. These results will provide references for the cloning and molecular breeding of low-nitrogen tolerance gene of rice.

## Materials and Methods

### Plant Materials

The 295 rice varieties from Northeast Agricultural University were adopted in this experiment. Plump seeds were kept at incubator at 48 °C for 48 h to break their dormancy. Then sixteen seeds of each accession were sowed in one row of two 96-well PCR plates respectively with one seed in one well. The seeds were soaked and sterilized in 1% sodium hypochlorite solution for 30 min. After 3 times of washing with distilled water, the sterilized seeds were placed in incubator at 31 °C and germinated in the following 2 days. The germinated seeds were cultured in the greenhouse (23.8 °C/22.4 °C, 10 h in the day/14 h at night). NH_4_NO_3_ was chosen as nitrogen source. The low and high nitrogen treatments were adopted as 8 ppm and 40 ppm, respectively. Firstly, sixteen seedling of each accession were cultured under high-nitrogen treatment (40 ppm) for 5 days; then, eight seedlings were kept in the same solution for 21 days, while the other eight seedlings were transplanted into low-nitrogen treatment (8 ppm) for 21 days. Hydroponic nutrient solution was prepared by referring to IRRI's conventional nutrient solution formula (Xin et al. 2019). The nutrient solution was replaced every seven days.

### Phenotype Data

Root length (RL) and root diameter (RD) were measured by LA-S plant root analysis system (Microtek Scan Makeri800) after 21 days of treatment. The above-ground and underground parts of seedlings were harvested and dried in oven at 105 °C for 30 min, respectively. After the samples were dried to constant weight, their dry matter weight was measured. Nitrogen content in aboveground and underground parts was analyzed by an element analyzer (Primacs SNC 100-IC-E), and nitrogen accumulation were calculated. The relative value of each trait was represented by the quotient of phenotypic value under low-nitrogen treatment divided by the value under high-nitrogen treatment.

### GWAS

Plink 2.0 software (Chang et al. 2015) was used to screen 788,396 SNPs developed by re-sequencing 295 rice varieties (Li et al. 2019). GWAS was conducted via the mixed linear model (MLM) of Tassel 5.0 (Bradbury et al. 2007). The threshold of SNP significantly associated with trait was set as *P* < 5.46 × 10^−6^. The Manhattan map was drawn by the CMplot package of R (Yin 2017). In order to identify the leading SNPs with the lowest *P*-value, redundant SNPs were filtered in a least-distance interval. The annotation information of the genes in the QTL interval was checked from the ensembl genome database (https://plants.ensembl.org/). The pairwise R^2^ value between any two SNPs within the interval of leading SNPs ± 2 Mb were calculated by LDBlockShow software (Dong et al. 2021). The average of top 10% R^2^ value within the interval from leading SNPs + 1.5 Mb to leading SNPs + 2 Mb was recorded as the background value of linkage disequilibrium (LD) attenuation. The background value of LD attenuation plus 0.2 was adopted to define the LD attenuation interval of the leading SNPs.

### RNA-Seq

**Fig. 10:**
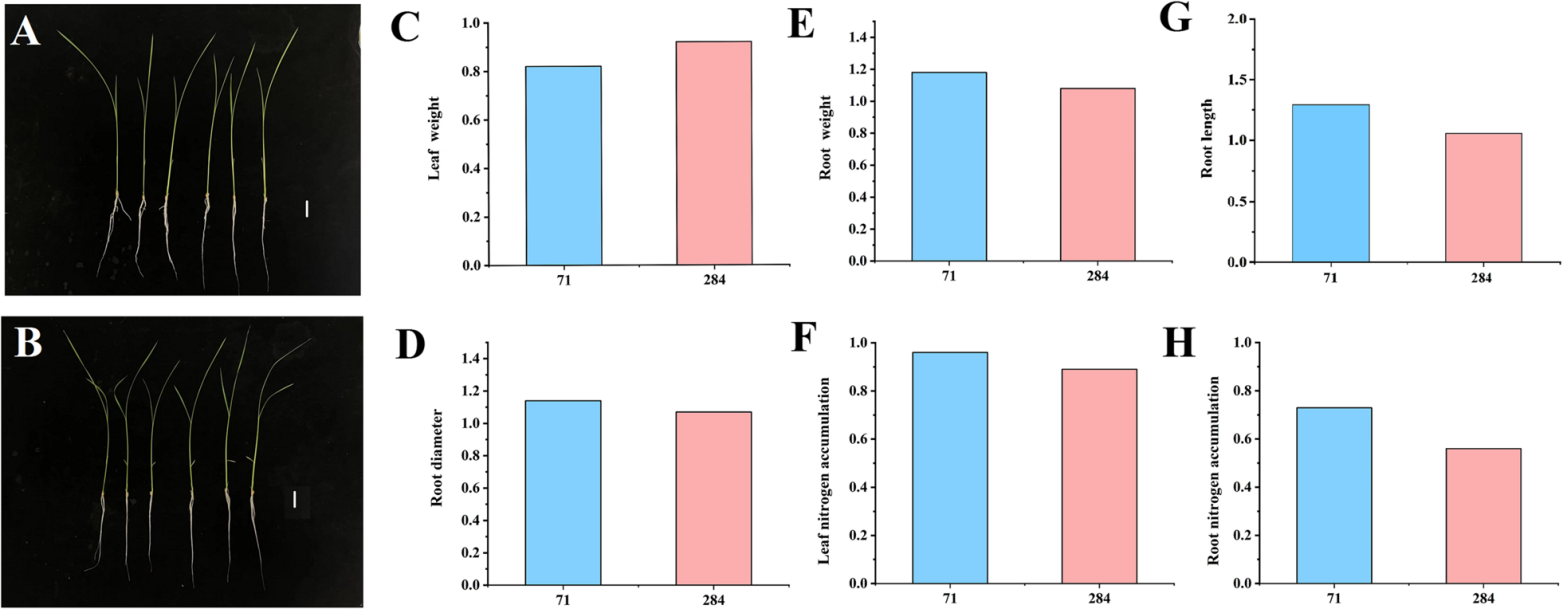
Root morphology traits of low-nitrogen tolerant variety (Longjing 31) and low-nitrogen sensitive variety (Songjing 10) under low and high nitrogen treatments. **A** The seedling of Longjing 31 under high (three on the left) and low (three on the right) nitrogen treatments. **B** The seedling of Songjing 10 under high (three on the left) and low (three on the right) nitrogen treatments. Scale bar represented 2 cm. **C** The relative value of the leaf weight under low and high nitrogen treatments. **D** The relative value of the root diameter under low and high nitrogen treatments. **E** The relative value of the root weight under low and high nitrogen treatments. **F** The relative value of the leaf nitrogen accumulation under low and high nitrogen treatments. **G** The relative value of the root length under low and high nitrogen treatments. **H** The relative value of the root nitrogen accumulation under low and high nitrogen treatments. 71: Longjing 31. 284: Songjing 10

According to phenotype data of 295 rice accessions, Longjing 31 and Songjing 10 were selected as low-nitrogen tolerant variety and low-nitrogen sensitive variety, respectively (Additional file 9: Table S8 and Fig. 10). Firstly, sixteen seedling of each variety (Longjing 31 and Songjing 10) were cultured under high-nitrogen treatment (40 ppm) for 5 days; then, eight seedlings were kept in the same solution for 7 days, while the other eight seedlings were transplanted into low-nitrogen treatment (8 ppm) for 7 days. After 12 days, 12 root samples (three replicates of each treatment) were collected. Total RNA was extracted from the 12 samples using the TransZol Up RNA Kit (TransGen Biotech, Beijing, China). Complementary DNA was synthesized from total RNA using the HiFiScript cDNA Synthesis Kit (CWBio, Beijing, China). An Illumina library was constructed according to the manufacturer’s instructions (Illumina, San Diego, CA, USA). High-throughput RNA sequencing was performed using the Illumina HiSeq 2500 platform. HISAT v2.1.0 was adopted to construct the index, and to map clean reads to the reference genome. The gene alignment and FPKM were calculate by using featureCounts v1.6.2 (Yang et al. 2014). The *P* < 0.05 and |log_2_FC|> 1 were adopt as the threshold to identify the differential expressed genes between any two comparative groups by edgeR v3.24.3 (Robinson et al. 2010).

### Quantitative Real-Time PCR

QRT-PCR analysis were performed to verify the expression levels of 12 genes selected randomly in Longjing 31 and Songjing 10 under low and high nitrogen treatments. Roche LightCycler96 was applied to conduct qRT-PCR following the manufacturers’ instructions. All primers were listed in Additional file 10: Table S9.

### Candidate Gene Prediction

The genes in the Leading SNP intervals were integrated to predict candidate genes. The haplotypes of candidate genes were screened and analyzed. Combined with the amount of gene expression, genes which expressed differentially in low-nitrogen tolerant variety (Longjing 31) were adopted to identify more valuable candidate genes.

### Data Analysis

The density distribution graph of each trait was plotted using “ggplot2” in R software. Phenotypic data were processed with IBM SPSS Statistics 26.0 software (SPSS Inc., Chicago, IL, USA.) for correlation analysis and descriptive statistical analysis, including mean, range and coefficient of variation.

## Supplementary Information

**Additional file 1: Figure S1.** Phenotype distribution of root morphology traits of 295 rice varieties under low and high-nitrogen treatments. A, HRL: root length under high-nitrogen treatment. B, LRL: root length under low-nitrogen treatment. C, HRD: root diameter under high-nitrogen treatment. D, LRD: root diameter under low-nitrogen treatment.

**Additional file 2: Table S1.** Descriptive statistics of phenotypic traits in natural populations.

**Additional file 3: Table S2.** Phenotype analysis of natural populations.

**Additional file 4: Table S3.** Significant associated loci.

**Additional file 5: Table S4.** Annotation and haplotype analysis of associated genes.

**Additional file 6: Table S5.** Summary of RNA-seq data.

**Additional file 7: Table S6.** RNA-seq data of the 14 differential expressed genes associated with nitrogen utilization.

**Additional file 8: Table S7.** RNA-seq data of 58 genes.

**Additional file 9: Table S8.** Phenotypic data after 21 days of treatment.

**Additional file 10: Table S9.** Sequences of primers used in this study.

## Data Availability

The datasets supporting the conclusions of this article are included within the additional files.

## Abbreviations

SNPs: Single nucleotide polymorphisms
QTL: Quantitative trait locus
DEGs: Differential expressed genes
RL: Root length
RD: Root diameter
RDR: The relative value of root diameter
RLR: The relative value of root length
HN: High-nitrogen treatment
LN: Low-nitrogen treatment
LRD: Root diameter under low-nitrogen treatment
HRD: Root diameter under high-nitrogen treatment
LRL: Root length under low-nitrogen treatment
HRL: Root length under high-nitrogen treatment
qRT-PCR: Real time quantitative polymerase chain reaction
InDel: Insertion deletion
BSA: Bulked segregant analysis
CDS: Sequence coding for amino acids in protein
