# Supplementary figures and images for: Mapping of Candidate Genes in Response to Low Nitrogen in Rice Seedlings
Source: Rice (N Y). 2022 Oct 15;15:51. doi: 10.1186/s12284-022-00597-x (PMC9569405; doi:10.1186/s12284-022-00597-x)

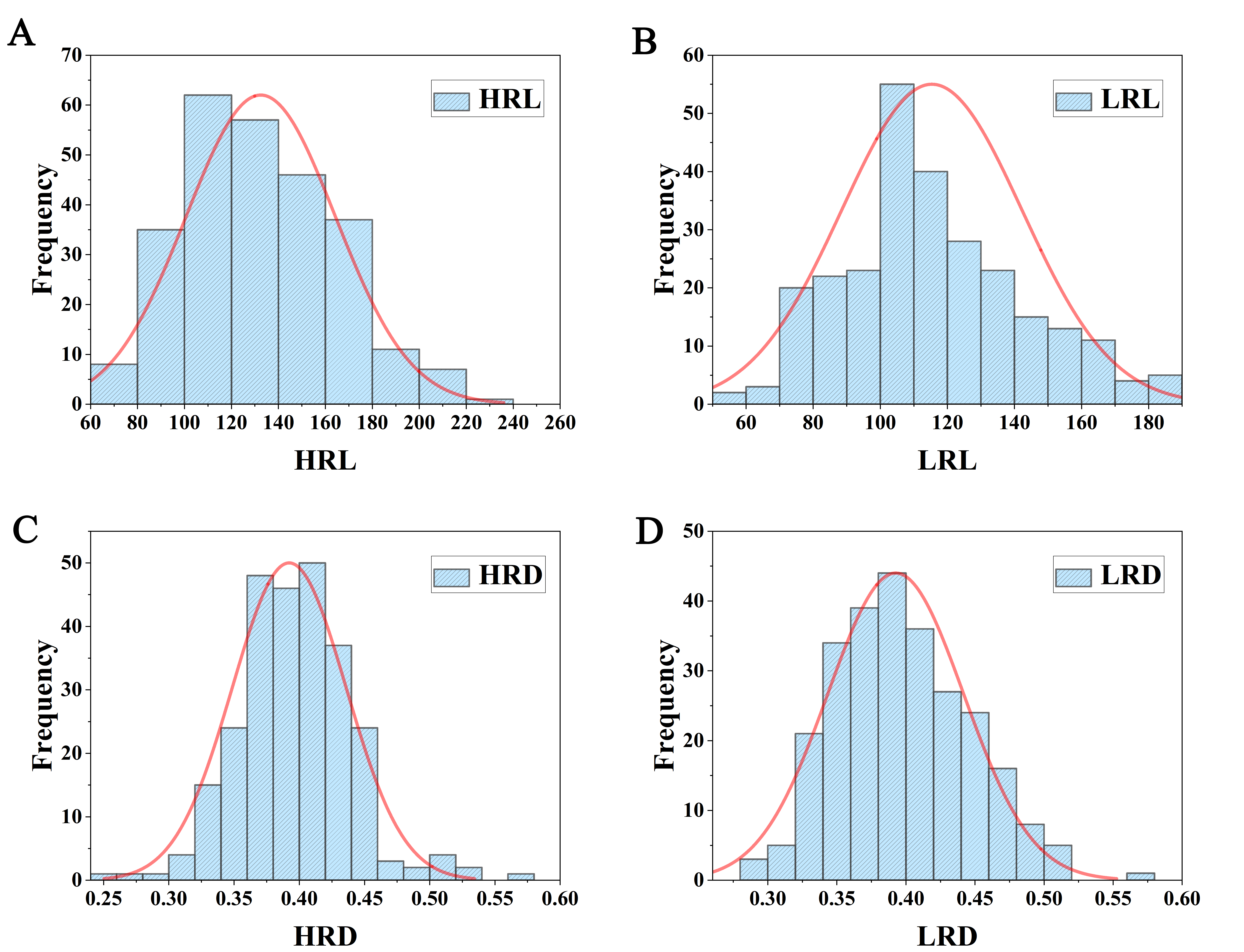

Supplement: Supplementary file 1 — Additional file 1: Figure S1. Phenotype distribution of root morphology traits of 295 rice varieties under low and high-nitrogen treatments. A, HRL: root length under high-nitrogen treatment. B, LRL: root length under low-nitrogen treatment. C, HRD: root diameter under high-nitrogen treatment. D, LRD: root diameter under low-nitrogen treatment. [file 12284_2022_597_MOESM1_ESM.tif]
